# Transcutaneous auricular vagus nerve stimulation in adult abdominal epilepsy associated with ketosis-prone diabetes: A case report

**DOI:** 10.1097/MD.0000000000049481

**Published:** 2026-06-26

**Authors:** Wei Zhang, Jingwei Ren, Guohai Li, Rundong Tang, Weiping Tian, Tongzhou Liang, Guohui Zhang, Junquan Liang

**Affiliations:** a Department of Acupuncture and Moxibustion, Shenzhen Bao’an Chinese Medicine Hospital, The Seventh Clinical Medical School of Guangzhou University of Chinese Medicine, Shenzhen, Guangdong, China; b The Second Clinical College of Guangzhou University of Chinese Medicine, Guangzhou, Guangdong, China; c Department of Acupuncture and Moxibustion, The Fourth Clinical Medical School of Guangzhou University of Chinese Medicine, Shenzhen Traditional Chinese Medicine Hospital, Shenzhen, Guangdong, China; d Department of Acupuncture and Moxibustion, Zhuhai Hospital of Integrated Traditional Chinese and Western Medicine, Zhuhai, Guangdong, China; e Institute of Biomedicine and Biotechnology, Shenzhen Institute of Advanced Technology, Chinese Academy of Sciences, Shenzhen, Guangdong, China; f Department of Geriatrics, Shenzhen Bao’an District People’s Hospital, Shenzhen, Guangdong, China; g The Brain Cognition and Brain Disease Institute (BCBDI), Shenzhen Institute of Advanced Technology (SIAT), Chinese Academy of Sciences (CAS), Shenzhen-Hong Kong Institute of Brain Science-Shenzhen Fundamental Research Institutions, Shenzhen, Guangdong, China.

**Keywords:** abdominal epilepsy, case report, diabetic ketoacidosis, drug-resistant epilepsy, ketosis-prone diabetes, transcutaneous auricular vagus nerve stimulation, type 2 diabetes mellitus

## Abstract

**Rationale:**

Abdominal epilepsy (AE) is a rare form of focal epilepsy that may present with recurrent paroxysmal gastrointestinal symptoms and can be difficult to distinguish from metabolic or gastrointestinal disorders. Ketosis-prone diabetes (KPD) may further complicate diagnosis because diabetic ketoacidosis, hypoglycemia, and glycemic instability can produce overlapping abdominal and neurobehavioral manifestations.

**Patient concerns:**

A 35-year-old Chinese man with poorly controlled type 2 diabetes mellitus and recurrent ketosis presented with severe abdominal pain and vomiting during an episode of diabetic ketoacidosis. He had a 2-year history of recurrent stereotyped periumbilical abdominal pain.

**Diagnoses:**

After unrevealing gastrointestinal, vascular, toxic, and metabolic evaluation, ictal electroencephalography (EEG) showed left temporal rhythmic sharp-slow-wave complexes with ipsilateral spread. The patient was diagnosed with ketosis-prone diabetes and EEG-supported AE, later considered drug-resistant.

**Interventions:**

Management included metabolic stabilization, oxcarbazepine–lamotrigine antiseizure therapy, and subsequent adjunctive transcutaneous auricular vagus nerve stimulation (taVNS) for persistent stereotyped abdominal episodes.

**Outcomes:**

During follow-up after adjunctive taVNS, the patient reported fewer episodes of abdominal pain. Diabetic ketoacidosis did not recur, and no device-related adverse events were observed. Because improvement occurred alongside continued antiseizure therapy and metabolic stabilization, causality cannot be inferred from this single-case observation.

**Lessons:**

In diabetic patients with recurrent unexplained abdominal pain, AE should be considered when episodes are stereotyped, abrupt, or accompanied by orofacial automatisms, altered awareness, autonomic instability, or a mismatch between symptom severity and abdominal findings. Early EEG may help reduce diagnostic delay, whereas adjunctive taVNS should be viewed as exploratory.

## 1. Introduction

Abdominal epilepsy (AE) is a rare form of focal epilepsy that can present predominantly with paroxysmal gastrointestinal symptoms and may therefore mimic common abdominal disorders. Diagnostic uncertainty may be further increased when AE occurs in patients with ketosis-prone diabetes (KPD), in whom metabolic instability can obscure seizure-related manifestations. Evidence regarding transcutaneous auricular vagus nerve stimulation (taVNS) in this combined neuro-metabolic setting remains limited. Here, we report an EEG-supported case of drug-resistant AE associated with KPD in which taVNS was introduced as an adjunct to ongoing antiepileptic therapy. This report focuses on the diagnostic challenge and the cautious interpretation of an exploratory adjunctive intervention.

## 2. Case report

### 2.1. Patient information

This case presentation was approved for exemption by the authors’ institutional review board, and consent was obtained from the patient.

On December 11, 2023, a 35-year-old man presented to the emergency department with severe, persistent, and poorly localized colicky abdominal pain accompanied by frequent vomiting of gastric contents. Symptoms began 15 minutes after ingestion of seafood. He denied diarrhea or hematochezia.

The patient had a 6-month history of type 2 diabetes mellitus (T2DM) with a positive family history of diabetes. He also had a 2-year history of recurrent paroxysmal periumbilical abdominal pain. He was nonadherent to his medication regimen (metformin 1.0 g twice daily; gliclazide modified-release tablets 30 mg once daily), resulting in poor glycemic control. He worked in stainless steel and aluminum installation, but denied any known exposure to toxic substances or drug allergies. His BMI was 26.6 kg/m^2^ (height 169 cm, weight 76 kg).

### 2.2. Clinical findings

Initial physical examination revealed mild epigastric tenderness and guarding but no rebound tenderness, suggesting a nonsurgical acute abdomen. Laboratory results revealed significant leukocytosis (WBC 16.64 × 10^9^/L; neutrophils 82.7%), pronounced hyperglycemia (17.2 mmol/L), and a fasting serum C-peptide level > 0.42 ng/mL. GAD65 antibodies were negative. Following symptomatic treatment (ondansetron, omeprazole, and trimebutine), the intractable vomiting subsided slightly. Despite persistent metabolic and inflammatory abnormalities, the patient left the hospital against medical advice.

He returned to the emergency department at 11:00 am the same day due to symptom exacerbation. Repeat laboratory investigations demonstrated persistent leukocytosis (WBC 17.17 × 10^9^/L; 90.5% neutrophils) and hyperglycemia (17.1 mmol/L). Arterial blood gas analysis indicated metabolic acidosis (pH 7.28; HCO_3_^–^14.8 mmol/L) with ketonemia (3.2 mmol/L). Urinalysis confirmed 3 + glucosuria and 3 + ketonuria.

A standardized DKA protocol was initiated with a regular insulin bolus (0.1 U/kg) followed by continuous infusion (0.1 U/kg/h). Blood glucose then declined rapidly (>4.2–5.6 mmol/L/h), accompanied by hypoglycemia and altered mental status. Insulin was temporarily suspended, and 25 mL of 50% dextrose was administered. Following stabilization (target 8.3–11.1 mmol/L), insulin was resumed at a reduced rate (0.02–0.05 U/kg/h) with dextrose supplementation.

Notably, the exacerbation of abdominal pain coincided temporally with the hypoglycemic episode. While adjusting the insulin regimen reduced pain intensity, the frequency of attacks persisted. Given the diagnostic uncertainty and unremarkable abdominal computed tomography findings, the patient was admitted.

Due to refractory abdominal pain, he was transferred to a tertiary center. Computed tomography angiography ruled out aortic dissection, and serum tumor markers were negative. A comprehensive gastrointestinal evaluation was unrevealing, prompting multidisciplinary team consultation. Lead poisoning was considered because of his occupational history, but blood lead levels were within normal limits. Psychogenic causes were considered only after exclusion of organic and metabolic etiologies. During this stage, venlafaxine (75 mg/day) and diazepam (2.5 mg/day) were initiated empirically for symptomatic stabilization while psychogenic causes remained in the differential diagnosis.

**Figure 1.**
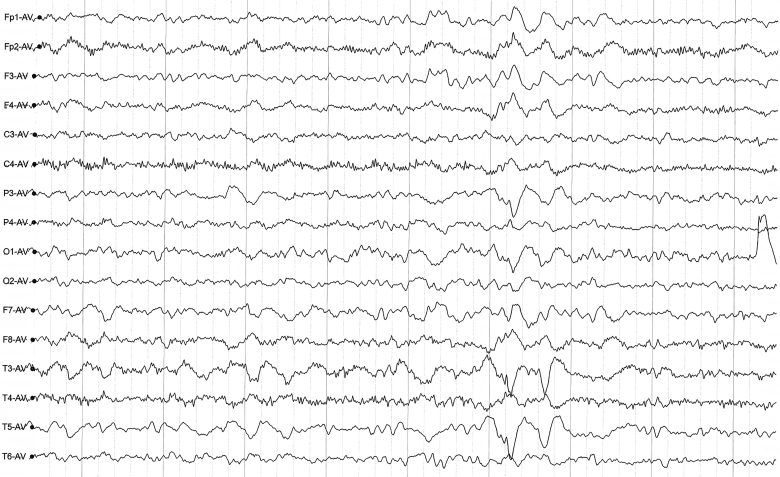
Ictal scalp EEG showing focal epileptiform discharges with left temporal predominance. Representative ictal scalp EEG recorded during an abdominal pain episode. High-amplitude rhythmic sharp-slow-wave complexes are maximal over the left temporal derivations (T3/T5), with extension to adjacent ipsilateral channels, supporting a focal ictal pattern. EEG = electroencephalography.

Electroencephalography (EEG) subsequently provided the key diagnostic evidence. Ictal EEG demonstrated rhythmic sharp-slow-wave complexes maximal over the left temporal region, with spread to the adjacent left parietal region (Fig. 1). Two weeks later, upon transfer to City Central Hospital, the patient again presented in acute distress with marked agitation. He described paroxysmal epigastric pain characterized by abrupt onset and offset, accompanied by nausea and retching. Vital signs indicated autonomic instability, including tachycardia (114 bpm) and hypertension (151/89 mm Hg). A significant dissociation was observed between symptom severity and physical findings; the abdomen was soft with only mild periumbilical tenderness and no peritoneal signs. Biochemical profiling confirmed persistent hyperglycemia (13.99 mmol/L) and ketonemia (3.1 mmol/L), while acute intermittent porphyria and pancreatitis were excluded by negative light-protected urine porphobilinogen testing and a normal serum lipase level, respectively.

**Figure 2.**
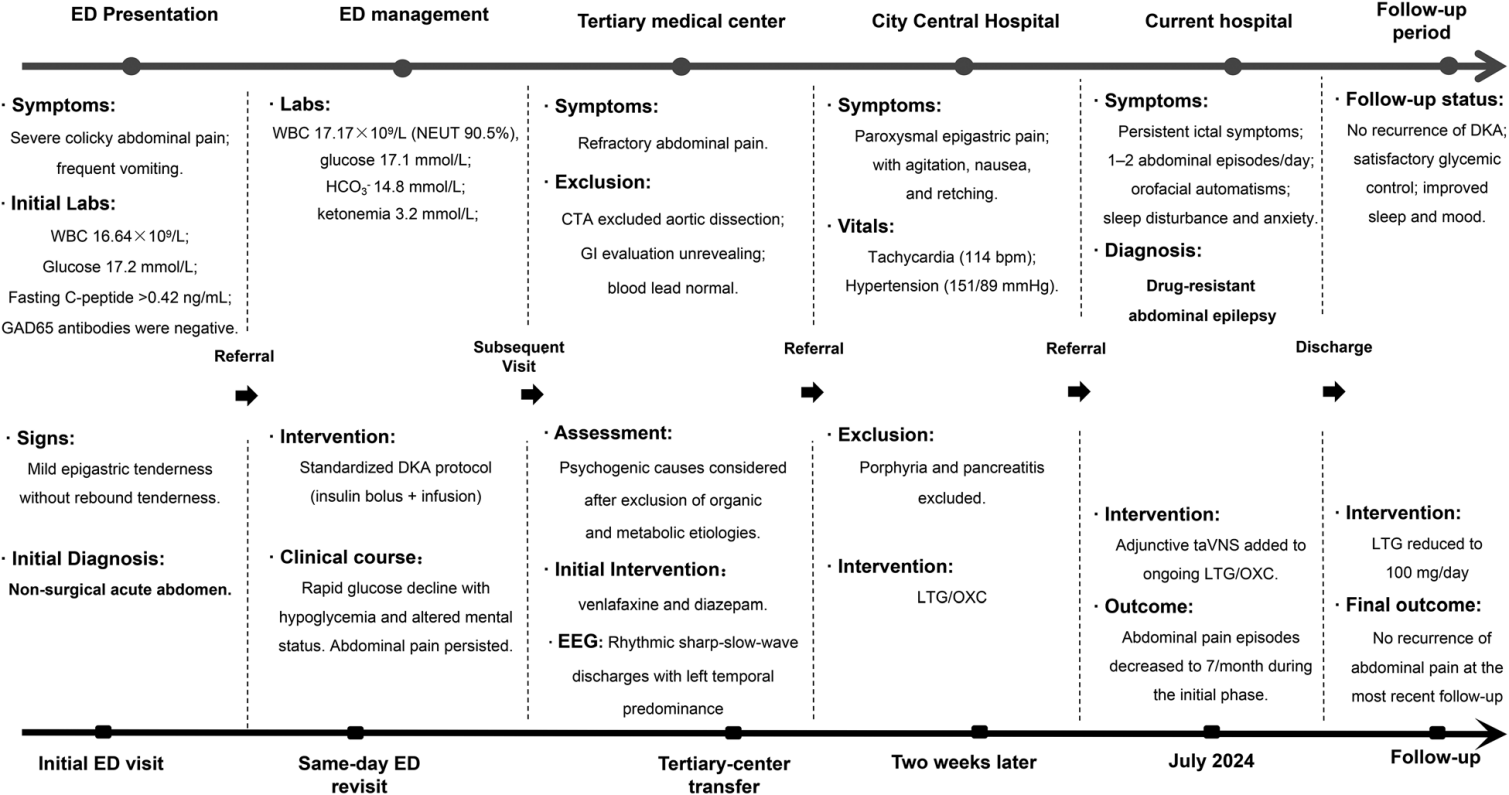
Timeline of the clinical course, diagnostic workup, treatment, and follow-up. The timeline summarizes the patient’s clinical course from the initial emergency department presentation to the most recent follow-up. The major events include severe recurrent abdominal pain with metabolic instability, same-day emergency department revisit with diabetic ketoacidosis and hypoglycemia during treatment, exclusion of gastrointestinal, vascular, toxic, porphyria-related, and pancreatic etiologies, ictal EEG-supported diagnosis of Abdominal epilepsy (AE), escalation of oxcarbazepine and lamotrigine therapy, diagnosis of drug-resistant abdominal epilepsy, initiation of adjunctive taVNS in July 2024, and follow-up outcomes including no recurrent diabetic ketoacidosis, satisfactory glycemic control, patient-reported improvement in sleep and mood, and no recurrent abdominal pain at the most recent follow-up. Bpm = beats per minute, CTA = computed tomography angiography, DKA = diabetic ketoacidosis, ED = emergency department, EEG = electroencephalography, GI = gastrointestinal, LTG = lamotrigine, NEUT = neutrophil percentage, OXC = oxcarbazepine, taVNS = transcutaneous auricular vagus nerve stimulation, WBC = white blood cell count.

A timeline of the clinical course is provided in Figure 2.

### 2.3. Therapeutic intervention

After ictal EEG findings supported an epileptic etiology, treatment focus shifted to dual antiseizure therapy with oxcarbazepine (OXC) and lamotrigine (LTG). OXC was initiated at 600 mg/day and titrated by 300 mg/week to a maintenance dose of 1200 mg/day in 2 divided doses. LTG was started at 25 mg/day and titrated by 50 mg every 2 weeks to a target dose of 200 mg/day. Serum sodium levels were monitored throughout treatment because of the risk of hyponatremia. Once the epileptic etiology had been established, venlafaxine was gradually tapered and discontinued.

Despite adherence to dual AED therapy (LTG and OXC), the patient exhibited persistent ictal symptoms. He reported an average of 1 to 2 episodes of spasmodic abdominal pain per day, each lasting 5 to 10 minutes and accompanied by focal neurological signs, specifically orofacial automatisms. Neuropsychiatric evaluation revealed marked mood disturbances, agitation, irritability, impaired awareness, and poor insight into his illness.

By July 2024, the patient had remained on LTG and OXC for several months, including sustained treatment after dose titration, yet adequate seizure control had not been achieved. Consequently, the clinical course was considered consistent with drug-resistant AE (DRE-AE). The persistent seizures, along with secondary sleep disturbances and severe anxiety, significantly impaired the patient’s quality of life.

Furthermore, the complexity of the multi-drug regimen may have contributed to reduced medication adherence, necessitating referral to our institution for specialized management.

After obtaining informed consent regarding potential risks and benefits, a combined intervention strategy was implemented. Transcutaneous auricular vagus nerve stimulation (taVNS) was introduced as an add-on therapy to the existing AED regimen. The patient commenced a 12-week taVNS treatment course using a Hwato TENS-200A stimulator (Suzhou Medical Appliance Factory, China). Following routine skin preparation with 75% medical ethanol, the dual-electrode system was applied to the ear. Specifically, the auricular electrode (5.8 × 11 mm) was attached to the concha, and the earplug electrode (9.8 × 12 mm) was positioned at the opening of the external auditory canal, effectively targeting the auricular branch of the vagus nerve (ABVN). Under physician guidance, the patient self-administered the treatment. The device was set to Mode B, delivering a dense-disperse wave alternating between 20 Hz for 7 seconds and 4 Hz for 3 seconds, with a constant pulse width of 0.2 ms. The stimulation intensity (ranging from 0 to 40 levels) was individually titrated to a distinct but non-painful sensory threshold, ensuring the maximum current remained below 50 mA (at a 500 Ω load impedance). Each treatment session lasted 30 minutes and was administered once daily. During the initial treatment phase, the frequency of paroxysmal abdominal pain episodes decreased to 7 per month compared with baseline.

### 2.4. Follow-up and outcomes

Upon completing the 12-week intensive phase, the patient entered regular follow-up and evaluation. Over the subsequent 3-month follow-up period, there were no recurrences of diabetic ketoacidosis (DKA), and glycemic control was maintained at a satisfactory level. The patient also reported subjective improvement in sleep quality and mood, which was consistent with follow-up clinical assessment. Given the stable clinical course, the LTG dose was reduced to 100 mg/day at 6 months after initiation of taVNS. At the most recent follow-up, no recurrence of abdominal pain had been reported.

Adherence to the intervention was assessed through patient self-reports and scheduled follow-up visits, during which the frequency and consistency of taVNS use were evaluated. Tolerability was assessed based on patient-reported outcomes and clinical observations, with particular attention to local discomfort, dizziness, and other potential device-related adverse effects. The patient tolerated taVNS well, with no adverse or unexpected events reported.

### 2.5. Patient perspective

In accordance with the CARE guidelines, the patient shared his subjective experience regarding the treatment course. He noted: “Before the ear stimulation, I was constantly in the hospital for stomach pain and felt exhausted all the time. Since starting the treatment, my stomach doesn’t hurt anymore, and I’m sleeping much better. I also feel less anxious, and my head feels clearer now that I’m taking less medication. It feels like I have my life back.”

## 3. Discussion

AE is a rare form of focal epilepsy characterized by recurrent paroxysmal gastrointestinal symptoms with epileptiform EEG abnormalities.^[1,2]^ The present case is clinically relevant because drug-resistant AE occurred in the context of KPD, creating substantial diagnostic overlap between seizure-related abdominal manifestations and metabolic disease. Reports describing adjunctive transcutaneous auricular vagus nerve stimulation (taVNS) in this combined neuro-metabolic setting remain extremely limited. Therefore, this case should be interpreted as a hypothesis-generating observation rather than evidence of treatment efficacy.

The diagnosis in this case depended on a stepwise process rather than on abdominal symptoms alone. Extensive gastrointestinal, vascular, toxic, and metabolic evaluation was unrevealing, whereas stereotyped paroxysms, orofacial automatisms, altered awareness, abrupt onset and offset, and autonomic instability increased suspicion of a seizure-related disorder. This clinical suspicion was strengthened by ictal EEG findings showing rhythmic sharp-slow-wave complexes maximal over the left temporal region with ipsilateral spread. Although diabetic ketoacidosis, hypoglycemia, and glycemic instability could have contributed to transient neurological or seizure-like manifestations, the recurrent stereotyped semiology, ictal EEG abnormalities, and persistence of episodes beyond isolated metabolic derangements argued against a purely metabolic explanation. Metabolic disturbances were therefore considered potential precipitating or aggravating factors, rather than the sole cause of the patient’s seizure-related manifestations.^[3–8]^

Because recurrent stereotyped episodes persisted despite sustained treatment with appropriately titrated oxcarbazepine and lamotrigine, the clinical course was considered consistent with the International League Against Epilepsy definition of drug-resistant epilepsy.^[9]^ Neurostimulation, including invasive vagus nerve stimulation, has been evaluated as an adjunctive approach for selected patients with drug-resistant epilepsy.^[10–13]^ Because invasive vagus nerve stimulation requires surgical implantation and device-related management, these considerations, together with the patient’s refractory clinical course, supported consideration of taVNS as a noninvasive exploratory adjunct, not as an established therapy for AE.^[14,15]^

Stimulation of the auricular branch of the vagus nerve may activate afferent projections to the nucleus of the solitary tract and downstream brainstem-thalamocortical networks implicated in seizure modulation.^[16–20]^ Emerging translational evidence further suggests that vagal neuromodulation may influence glycemic regulation, although the available evidence remains preliminary and mechanistically heterogeneous.^[21,22]^ Vagal neuromodulation has also been linked to autonomic, neurotransmitter, and neuroimmune pathways that may be relevant to seizure modulation.^[23,24]^ In this single patient, these mechanisms remain theoretical and should not be interpreted as evidence of causality or therapeutic efficacy. This case has practical diagnostic implications. In patients with diabetes who present with recurrent unexplained abdominal pain, EEG evaluation should not be considered routinely on the basis of metabolic instability alone. Rather, early neuroelectrophysiological assessment may be warranted when episodes are recurrent and stereotyped, show abrupt onset and offset, or are accompanied by automatisms, altered awareness, autonomic features, or a disproportion between symptom severity and abdominal findings. This case therefore supports keeping seizure-related disorders in the differential diagnosis when abdominal symptoms are episodic and neurologically patterned.^[3,25]^

Several limitations should be acknowledged. First, this was a single-case observation without a control condition; therefore, spontaneous fluctuation, placebo effects, concomitant clinical management, continued antiepileptic therapy, improved adherence, and improved metabolic control cannot be excluded. Second, outcome ascertainment was limited. Repeat EEG after taVNS was not available, seizure frequency was not prospectively recorded using a standardized seizure diary, and adherence was assessed mainly through patient self-report and scheduled follow-up visits. Patient-reported assessments were obtained during routine clinical follow-up rather than as predefined research outcomes, which limits the interpretation of these patient-centered changes. In this single case, adjunctive taVNS was temporally associated with improvement in seizure-related symptoms and patient-reported sleep and mood complaints; however, causality cannot be inferred from an uncontrolled single-case observation. Larger studies incorporating standardized seizure documentation, repeated electrophysiological assessment, validated patient-reported outcomes, and longer follow-up are needed to clarify the potential role and durability of taVNS in this neuro-metabolic context.

## Acknowledgments

The authors thank SciBio for English-language editing assistance.

## Author contributions

**Conceptualization:** Wei Zhang.

**Data curation:** Wei Zhang.

**Funding acquisition:** Rundong Tang, Junquan Liang.

**Investigation:** Jingwei Ren, Guohai Li, Rundong Tang, Weiping Tian, Tongzhou Liang.

**Project administration:** Guohui Zhang.

**Supervision:** Junquan Liang.

**Writing – original draft:** Wei Zhang.

**Writing – review & editing:** Wei Zhang, Junquan Liang.
